# Cerebrospinal Fluid C1-Esterase Inhibitor and Tie-1 Levels Affect Cognitive Performance: Evidence from Proteome-Wide Mendelian Randomization

**DOI:** 10.3390/genes15010071

**Published:** 2024-01-04

**Authors:** Loukas Zagkos, Marie-Joe Dib, Héléne T. Cronjé, Paul Elliott, Abbas Dehghan, Ioanna Tzoulaki, Dipender Gill, Iyas Daghlas

**Affiliations:** 1 Department of Epidemiology and Biostatistics, School of Public Health, Imperial College London, London SW7 2BX, UK; p.elliot@imperial.ac.uk (P.E.); a.dehghan@imperial.ac.uk (A.D.); i.tzoulaki@imperial.ac.uk (I.T.); dipender.gill@imperial.ac.uk (D.G.); 2 Division of Cardiovascular Medicine, Hospital of the University of Pennsylvania, Philadelphia, PA 19104, USA; marie-joe.dib@pennmedicine.upenn.edu; 3 Department of Public Health, Section of Epidemiology, University of Copenhagen, 1165 Copenhagen, Denmark; toinet.cronje@sund.ku.dk; 4 UK Dementia Research Institute at Imperial College London, Hammersmith Hospital, London W1T 7NF, UK; 5 Medical Research Council Centre for Environment and Health, School of Public Health, Imperial College London, London SW7 2AZ, UK; 6 Centre for Systems Biology, Biomedical Research Foundation of the Academy of Athens, 11527 Athens, Greece; 7 Department of Neurology, University of California, San Francisco, San Francisco, CA 94143, USA; iyas.daghlas@ucsf.edu

**Keywords:** CSF, cognition, tyrosine-protein kinase receptor, *SERPING1*, *TIE1*

## Abstract

Objective: The association of cerebrospinal fluid (CSF) protein levels with cognitive function in the general population remains largely unexplored. We performed Mendelian randomization (MR) analyses to query which CSF proteins may have potential causal effects on cognitive performance. Methods and analysis: Genetic associations with CSF proteins were obtained from a genome-wide association study conducted in up to 835 European-ancestry individuals and for cognitive performance from a meta-analysis of GWAS including 257,841 European-ancestry individuals. We performed Mendelian randomization (MR) analyses to test the effect of randomly allocated variation in 154 genetically predicted CSF protein levels on cognitive performance. Findings were validated by performing colocalization analyses and considering cognition-related phenotypes. Results: Genetically predicted C1-esterase inhibitor levels in the CSF were associated with a better cognitive performance (SD units of cognitive performance per 1 log-relative fluorescence unit (RFU): 0.23, 95% confidence interval: 0.12 to 0.35, *p* = 7.91 × 10^−5^), while tyrosine-protein kinase receptor Tie-1 (sTie-1) levels were associated with a worse cognitive performance (−0.43, −0.62 to −0.23, *p* = 2.08 × 10^−5^). These findings were supported by colocalization analyses and by concordant effects on distinct cognition-related and brain-volume measures. Conclusions: Human genetics supports a role for the C1-esterase inhibitor and sTie-1 in cognitive performance.

## 1. Introduction

Cerebrospinal fluid (CSF) is an ultrafiltrate of plasma produced in the cerebral ventricles and located within the subarachnoid space where it maintains the structural and physiological integrity of the central nervous system (CNS) [1,2]. The composition of CSF, including protein levels, provides information about brain health and may help to stratify neurodegenerative disease risk [3,4,5,6]. Given its proximity to nervous system tissue, CSF may be more informative about protein–disease relationships than serum. These proteins may also represent targets for the development of therapeutics that prevent or treat disease [7].

Prior studies have mostly focused on the association of CSF protein levels with the risk of dementia [5,6], while investigations into the relationship between CSF proteins and cognition more broadly have been limited [8]. For example, prior epidemiologic studies investigated the association between CSF biomarkers and cognitive function. The proteins investigated in these studies were curated based on known relationships with AD (e.g., amyloid beta, tau, and neurofilament light chain) [9,10,11]. Thus, the relationship of the proteome with cognition remains a largely unexplored frontier.

While epidemiologic studies contribute important information to the understanding of the association between biomarkers and disease, associations from conventional epidemiologic analyses can be biased by residual confounding or reverse causality and may therefore not represent causal relationships. Mendelian randomization (MR) is an alternative analytic paradigm that uses human genetic data to estimate less biased causal effects. MR leverages randomly allocated genetic variants as proxies for exposures to estimate the causal effect of an exposure (e.g., levels of a protein in the CSF) on an outcome (e.g., cognitive function) [12,13]. Due to the random and fixed allocation of genetic variants at conception, this approach is less susceptible to potential bias due to confounding from environmental factors or reverse causation. The recent discovery of genetic variants associated with CSF protein levels and the availability of large-scale genetic association studies of cognition-related phenotypes permits the application of MR to examine the relationship between the CSF proteome and cognitive performance [14]. In this work, we applied MR to systematically query the human CSF proteome for proteins with evidence for a causal effect on cognitive performance.

## 2. Materials and Methods

### 2.1. Study Design

**Figure 1:**
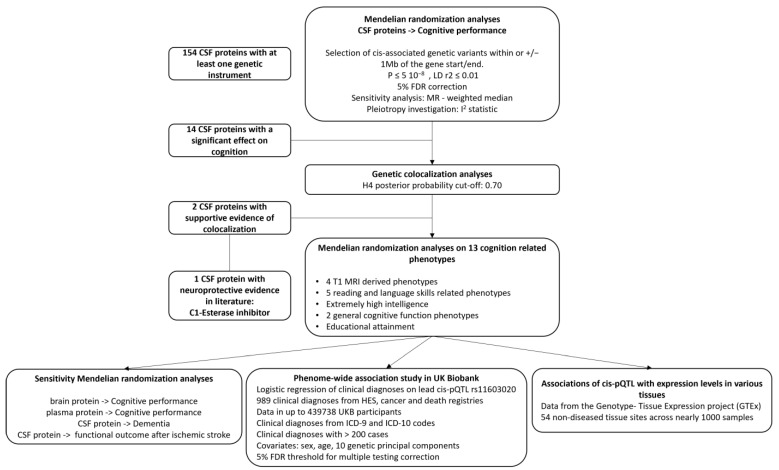
Analysis pipeline. Mendelian randomization (MR) analyses were performed to explore the association of genetically proxied levels of cerebrospinal fluid (CSF) proteins on cognitive performance. After a 5% false discovery rate (FDR) correction, 14 proteins that were significantly associated with cognitive performance were taken forward for genetic colocalization analysis. Two CSF proteins had evidence of genetic colocalization with cognitive performance and were included in an additional MR aimed at assessing their association with 13 complementary cognition-related phenotypes. Because of the literature-based support for the associations we observed with the CSF C1-esterase inhibitor, we conducted additional sensitivity and exploratory analyses including a phenome-wide association study for the lead CSF C1-esterase inhibitor *cis*-protein quantitative trait locus (pQTL) and gene expression association analysis.

The overall study design is provided in Figure 1. We conducted *cis*-Mendelian randomization (*cis*-MR) analyses to explore the associations of genetically predicted CSF protein concentration with cognitive performance. To ensure that MR findings were not biased by linkage disequilibrium (LD) with a nearby genetic association signal, further statistical validation was performed using genetic colocalization analyses [15]. For proteins with evidence from colocalization analyses, we conducted complementary MR analyses to assess the association of genetically predicted protein levels with 13 curated cognition-related phenotypes. This study is reported using the Strengthening the Reporting of Observational Studies in Epidemiology using Mendelian Randomization (STROBE-MR) guidelines (Supplementary Table S1).

### 2.2. Genetic Associations with Protein Levels in CSF and Other Tissues

Yang et al. conducted a genome-wide association study (GWAS) of 713 cerebrospinal fluid (CSF) proteins in 971 individuals recruited from the Washington University School of Medicine in St. Louis, including 249 patients with Alzheimer’s disease (AD), 717 cognitively normal controls, and 5 with unknown cognitive function status [14]. The average age of the study participants was 69 years (standard deviation [SD] 9.3 years), 53% were women, and 39% had at least one copy of the *APOE* ε4 allele. Study participants were unrelated and of European ancestry (detailed information available in the GWAS manuscript). After quality control by Yang et al., the total sample size was 835 patients. Of note, the authors demonstrated that genetic associations with protein levels were nearly identical between patients with and without AD and thus the inclusion of AD cases does not bias the genetic associations with protein levels (r > 0.98 for genetic associations with proteins in participants with and without AD). CSF was collected via lumbar puncture after an overnight fast. A multiplexed aptamer-based platform developed by SomaLogic Inc. (Boulder, CO, USA) was used to measure the relative concentrations of proteins in the CSF [16]. The protein levels were quantified as relative fluorescence units (RFU). Genetic association estimates with CSF protein levels were adjusted for sex, age, the first two genetic principal components, and the genotyping platform. In additional sensitivity analyses, we used genetic association data for plasma (n = 529) and brain (n = 380; measured using parietal cortex tissue) proteins from the same study.

### 2.3. Genetic Associations with the Outcomes

The primary outcome of this MR analysis was cognitive performance. We obtained genetic association estimates for cognitive performance from a Social Science Genetic Association Consortium (SSGAC) meta-analysis conducted by Lee et al. [17], including 257,841 European-ancestry UK Biobank [18] and Cognitive Genomics Consortium (COGENT) participants [19]. In the UK Biobank, cognitive performance was measured in 222,543 participants using a standardized score of a test of verbal–numerical reasoning. The test contained thirteen logic and reasoning questions with a two-minute time limit and was designed as a measure of fluid intelligence. Each respondent took the test up to four times, and the mean of the standardized scores was used, which was then standardized. In COGENT, cognitive performance was quantified as the first unrotated principal component of performance on at least three neuropsychological tests or two intelligence quotient (IQ) test subscales, measuring the overall accuracy or total number of correct responses in 35,298 individuals. In general, the test variable used either the overall accuracy or total number of correct responses. Adjustments for age, sex, and population stratification were included in the genotype–phenotype association analysis for each cohort.

In secondary analyses, we considered 13 cognition-related phenotypes as outcomes, to explore how proteins might impact different dimensions of cognitive function. Summary statistics were obtained for volumetric brain magnetic resonance imaging (MRI)-derived phenotypes (T1; left and right hippocampal volume, cortical gray matter volume, and total brain volume) in 33,224 UK Biobank participants [20]. Genetic associations with phenotypes related to reading and language skills (word reading, nonword reading, spelling, phoneme awareness, and nonword repetition) in up to 27,180 European-ancestry participants from a published GWAS meta-analysis [21]. Nonword reading and repetition are language assessment tasks where individuals are asked to read and repeat words that do not exist in the language lexicon, which are standard tests that can be used to identify individuals with language difficulties. Genetic association data were also obtained for a binary phenotype of ‘extremely high intelligence’ in 9410 individuals of European ancestry (1238 cases with a mean IQ > 170 and 8172 controls) [22]. For general cognitive function phenotypes, we included reaction time (N = 330,069) and verbal numeric reasoning (N = 168,033) from a GWAS meta-analysis by Davies et al. [23]. Finally, for educational attainment we made use of the meta-analysis by Lee et al. (N = 766,345) [17]. All secondary phenotypes are in standard deviation units apart from the MRI traits, which have mm^3^ units. There was no sample overlap between the exposure (CSF protein) and any of the outcome GWAS datasets.

### 2.4. Selection of Genetic Proxies for CSF Protein Levels

For MR analysis, we used *cis* protein quantitative trait loci (pQTLs) as genetic instruments for each CSF protein. Genetic variants were considered *cis* to the gene when they were located within 1 Mb of the gene start or end position. This decision to use *cis* variants was motivated by the notion that such variants are less likely to be biased when used to proxy protein levels compared to variants from throughout the genome (also referred to as trans variants; 7). Gene coordinates were determined based on the Genome Reference Consortium Human version 37 (GRCh37) released by the Ensembl genome browser. We selected genome-wide significant pQTLs (*p* < 5 × 10^−8^) that were also present in the outcome datasets. To obtain independent genetic instruments for each CSF protein, the variants were clumped using a pair-wise LD r^2^ < 0.01 (7) from the 1000 genomes project phase 3 European LD reference panel [24]. A distance-based threshold of 1 Mb was used as the clumping window.

### 2.5. Statistical Analysis

#### 2.5.1. Mendelian Randomization Analysis

We conducted two-sample MR analyses to explore the effect of CSF protein levels on the primary and secondary outcome phenotypes. Genetic associations with the exposures and outcomes were harmonized by aligning effect alleles, using the ‘TwoSampleMR’ v.0.6.0 R package [25]. MR effects were estimated either using the Wald ratio for single variant instruments [25], or the random-effects inverse-variance weighted method for instruments comprising multiple genetic variants [13]. MR effect estimates were considered statistically significant below a false discovery rate (FDR) of 5% to account for the multiple testing of correlated phenotypes. MR effect estimates are reported as the SD change in cognitive performance per 1-log RFU increase in the genetically predicted CSF protein level.

Where more than two genetic instruments were available for a given protein, we performed sensitivity analyses to assess the robustness of MR estimates to bias due to horizontal pleiotropy. Horizontal pleiotropy refers to the association of the genetic instrument with the outcome through pathways independent of the exposure and leads to potential bias in the MR estimate [26]. We first calculated the I^2^ statistic for heterogeneity, which is a global indicator of either heterogeneity or pleiotropy [27]. We then used the weighted median method which orders the MR estimates obtained by each genetic instrument by their magnitude, weighted for their precision, and calculates an overall MR estimate based on the median value [28]. Proteins with consistent MR estimates using the weighted median method were carried forward for colocalization analysis.

#### 2.5.2. Genetic Colocalization Analysis

Due to linkage disequilibrium, genetic variants in close proximity tend to be inherited together and are often correlated. If distinct, correlated causal variants are associated with the exposure and the outcome, this may introduce bias in the *cis*-MR effect estimates as it allows an association between a genetic variant and the outcome via an alternative pathway that does not pass through the exposure (violation of the exchangeability assumption). Genetic colocalization analysis is a Bayesian method that can be used as a sensitivity analysis to identify this type of genetic confounding [15]. This analysis determines whether the genetic associations for any given traits share the same causal variant. We implemented the ‘coloc’ method [29], which works under the assumption that there is no more than one causal variant per trait and compares evidence for different hypotheses in a Bayesian framework. The coloc algorithm returns posterior probabilities for five hypotheses, with the posterior probability H4 corresponding to the hypothesis of a shared causal variant in the genomic region (evidence of colocalization). The posterior probabilities were calculated from the prior probabilities (we used the ‘coloc’ R package (v.5.1.0.1) [30] default settings: p_1_ = 10^−4^, p_2_ = 10^−4^, p_12_ = 10^−5^). We applied an H4 posterior probability cut-off of 0.70 [31] but considered associations with lower posterior probabilities in exploratory analyses.

#### 2.5.3. Complementary Analysis for C1-Esterase Inhibitor

We performed follow-up analyses to better understand the effect of the C1-esterase inhibitor, which was the protein with the strongest supporting evidence of an association with cognitive performance from our analyses. To assess tissue specificity of the (CSF) pQTL, we assessed the association of genetically proxied levels of the C1-esterase inhibitor in plasma and brain tissue on cognitive performance. Second, given preclinical data suggesting neuroprotective effects of the C1-esterase inhibitor, we explored the associations of genetically predicted C1-esterase inhibitor levels in CSF with Alzheimer’s disease (35,274 AD cases, and 59,163 non-AD controls) [32], and with functional outcomes 3 months after ischemic stroke (N = 6021, modified Rankin scale) [33]. Third, to identify additional disease associations across a wide range of clinical diagnoses, we performed a phenome-wide association study (PheWAS), using the lead *cis*-pQTL of CSF C1-esterase inhibitor, rs11603020, in the UK Biobank. We used the International Classification of Diseases (ICD) versions 9 and 10 [34] to identify cases for the clinical health outcomes from the Hospital Episode Statistics (HES), cancer, and death registries. Clinical diagnoses were mapped to the phecode grouping system, using the ‘PheWAS’ R package v0.99.5.5 [35]. In PheWAS, we performed a series of logistic regressions using the effect allele (C allele) of rs11603020 as the exposure and each clinical diagnosis as an outcome in up to 439,738 UK Biobank participants of European ancestry. Age, sex, and the first 10 genetic principal components were included as covariates. To maintain statistically meaningful calculations, the analysis was limited to health outcomes for which we had sufficient power (N ≥ 200 cases) [36]. A 5% FDR threshold was used to define statistically significant associations. Finally, we explored the association of rs11603020 with gene expression in 54 healthy tissue sites across nearly 1000 samples from the Genotype-Tissue Expression project (GTEx) version 9 [37].

## 3. Results

**Figure 2:**
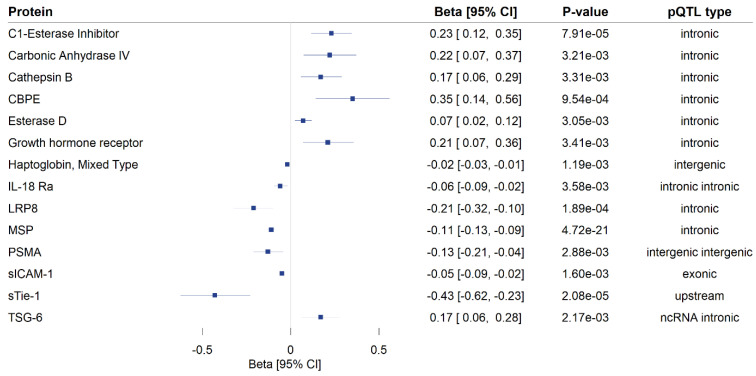
Mendelian randomization findings for the associations of genetically proxied cerebrospinal fluid (CSF) protein levels with cognitive performance. Only significant associations (*p* FDR < 0.05) are shown. MR estimates are reported as the standard deviation change in cognitive performance per 1 log-relative fluorescence unit (RFU) higher genetically predicted CSF protein level. The type of protein quantitative trait loci used as instruments in the analysis is provided in the fourth column. CI: confidence interval, pQTL: protein quantitative trait locus, ncRNA intronic: intronic non-coding RNA variants, CBPE: carboxypeptidase E, IL-18 Ra: interleukin-18 receptor 1, LRP8: low-density lipoprotein receptor-related protein 8, MSP: hepatocyte growth factor-like protein, PSMA: glutamate carboxypeptidase 2, sICAM-1: intercellular adhesion molecule 1, sTie-1: tyrosine-protein kinase receptor Tie-1, TSG-6: tumor necrosis factor-inducible gene 6 protein.

**Figure 3:**
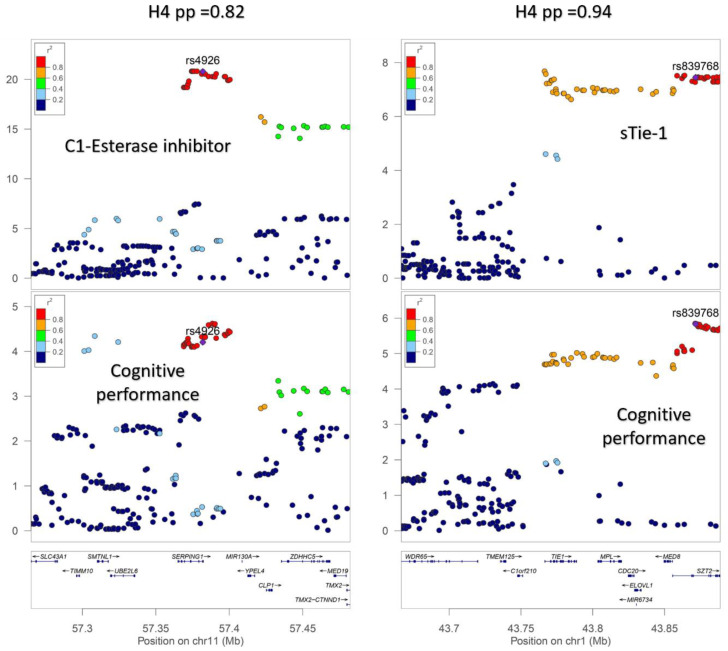
Regional association plots demonstrating colocalization between genetic association signals for cerebrospinal fluid (CSF) protein levels and cognitive performance. (I) Genetic associations with CSF C1-esterase inhibitor concentrations and cognitive performance (−log10(P)) plotted against chromosome position (megabases) for variants *cis* to *SERPING1*. (II) Genetic associations (−log10(P)) plotted against chromosome position (megabases) for variants within the *TIE1* gene or +/−100 kb of the gene start and end position for cerebrospinal fluid sTie-1 levels and cognitive performance. H4 pp: Posterior probability of colocalization.

A total of 172 genome-wide significant *cis*-pQTLs were used to instrument 154 CSF proteins in MR analysis, of which, 139 proteins had one instrument, 12 proteins had two, and three proteins had three instruments (Supplementary Table S2). Genetically predicted levels of 14 CSF proteins were significantly associated with cognitive performance (Supplementary Table S3), after using a 5% FDR statistical significance threshold (*p* < 6 × 10^−3^; Figure 2). We performed colocalization analyses to assess whether these associations are due to a shared causal genetic variant rather than genetic confounding through a neighboring variant in LD (Supplementary Table S4; Figure 3). Genetic determinants of two CSF proteins (C1-esterase inhibitor and sTie-1) colocalized with variants that determine cognitive performance, with a posterior probability for colocalization > 0.7. For sTie-1, rs839768 was identified to be the shared causal variant with cognitive performance with PPH4 = 0.94, whereas, for the C1-esterase inhibitor, rs4926 (p.Val480Met, a missense variant in *SERPING1*) was identified as the most probable shared causal variant with cognitive performance (PPH4 = 0.82). Although not meeting our prespecified threshold, variants related to LRP8 had suggestive evidence for colocalization with cognitive performance (PPH4 = 0.46) with an inverse association with cognitive performance in MR analyses (Supplementary Table S3). The lead variant used for analysis (rs12031155) was highly correlated (r^2^ 0.98) with rs5174, a missense variant in LRP8.

**Figure 4:**
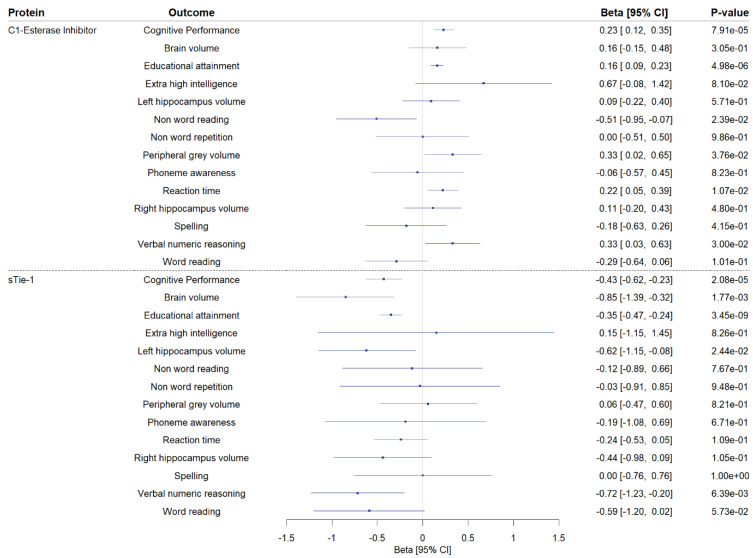
Secondary Mendelian randomization analyses investigating the association of genetically proxied cerebrospinal fluid (CSF) C1-esterase inhibitor and sTie-1 with phenotypes related to cognitive performance. Two-sample Mendelian randomization (MR) findings exploring the association of genetically proxied cerebrospinal fluid C1-esterase inhibitor and sTie-1 levels with cognitive performance and 13 cognitive performance-related phenotypes. MR estimates on outcomes are reported as the standard deviation change per 1 log-RFU higher genetically predicted CSF protein level, except for T1 MRI phenotypes (brain volume, left and right hippocampus volume, peripheral gray matter volume), where MR estimates are reported as mm^3^ change per 1 log-RFU higher genetically predicted CSF protein level, and for extremely high intelligence, where MR estimates are reported as log-odds change per 1 log-RFU higher genetically predicted CSF protein level. CI: confidence interval.

We conducted two-sample MR to explore the associations of genetically proxied CSF levels of sTie-1 and the C1-esterase inhibitor with phenotypes related to cognitive performance (Figure 4; Supplementary Table S5). Higher genetically predicted CSF C1-esterase inhibitor levels were associated with increased educational attainment, and nominally with a greater peripheral grey volume, faster reaction time, and improved verbal numeric reasoning. Higher genetically predicted levels of CSF sTie-1 were associated with a lower brain volume, lower educational attainment, and worse verbal numeric reasoning, and nominally with the lower left hippocampal volume.

In follow-up analyses, we found that 1 log-RFU higher genetically predicted plasma C1-esterase was associated with a 0.16 SD higher cognitive performance (beta: 0.16, 95% CI: 0.08 to 0.23, *p* = 4.73 × 10^−5^) (Supplementary Table S6). There was no evidence for an association of genetically proxied CSF C1-esterase inhibitor with the risk of AD or with functional outcomes three months after ischemic stroke (Supplementary Table S7). There were no PheWAS associations that met our statistical significance threshold. We identified a nominal association between the instrumented levels of CSF C1-esterase inhibitor and the odds of having a ‘rash and other nonspecific skin eruption’ (odds ratio [OR]: 1.13, 95% CI: 1.05 to 1.22, *p* = 8.49 × 10^−4^) in keeping with the known clinical syndrome of C1-esterase inhibitor deficiency (Supplementary Table S8). Finally, using gene-expression data, we found that rs10603020 influences the gene expression of *SERPING1* (the gene coding for the C1-esterase inhibitor) in whole blood (*p* = 5.3 × 10^−8^) (Supplementary Table S9).

## 4. Discussion

We queried the effects of 154 genetically predicted CSF protein levels on cognitive performance using the MR paradigm and found multiple lines of evidence to support the C1-esterase inhibitor and sTie-1 as proteins with relevance to cognitive performance. First, genetically predicted levels of these proteins were associated with cognitive performance in MR analyses. Second, these associations were further supported by colocalization analyses supporting a shared causal variant underlying the association signal between the CSF protein level and cognitive performance. In the case of the C1-esterase inhibitor, the prioritized shared variant was a coding variant in *SERPING1*, and such variants are generally considered to provide stronger evidence for support of gene–phenotype relationships (7). Third, these associations were supported by concordant associations with related cognition and neuroimaging phenotypes.

The C1-esterase inhibitor functions as a serine protease inhibitor involved in regulating the complement system activation to prevent an excessive immune response [38]. The C1-esterase inhibitor also participates in the regulation of the kallikrein–kinin system, which is involved in vascular tone and permeability, as well as the coagulation cascade [39]. These functions underlie its relevance in hereditary angioedema. In terms of brain health, the C1-esterase inhibitor has been investigated using animal models. These predominantly mouse studies have demonstrated that knockdown or inhibition of the C1-esterase inhibitor worsens cognition and neurovascular impairment [39], and the infusion of a recombinant C1-esterase inhibitor mitigates ischemic brain injury [40] and inflammation after traumatic brain injury [41]. Taken together with results from our genetic analyses, these preclinical data further support the hypothesis that the C1-esterase inhibitor may have neuroprotective properties in humans.

In contrast to the C1-esterase inhibitor, sTie-1 is poorly characterised in the literature. This protein has been implicated in various vascular processes such as angiogenesis and vessel wall integrity [42]. These processes have relevance to neural development and cerebrovascular disease and may mediate the association of sTie-1 with cognitive performance. Moreover, the sTie-1 lead *cis*-variant rs3768046 has been reported to be associated with the risk of attention deficit hyperactivity disorder (ADHD) via the dysregulated expression of *TIE1* [43].

Although the colocalization evidence for *LRP8* did not meet our predetermined statistical threshold, it is worth discussing this protein in the context of its known relevance to human brain health. Low-density lipoprotein receptor-related protein 8 (LRP8) is also known as apolipoprotein E receptor 2. The interaction of this receptor with the reelin ligand has been extensively studied for its role in neuronal migration and positioning during cortical development in mice [44]. The rs5174 variant in LRP8, which is highly correlated with the pQTL instrument used in the present analysis, has been robustly associated with the GWAS of brain cortical thickness and sulcal depth [45]. Thus, it is likely that this protein is influencing cognition through pathways that are active during neurodevelopment. Moreover, other proteins with significant MR associations but a lack of colocalization evidence are worth exploring further in future studies, such as carbonic anhydrase IV, cathepsin B, or growth hormone.

There are several strengths to highlight from this study. First, we employed the MR paradigm, which is robust against potential biases resulting from confounding and reverse causation. Second, genetic colocalization analyses confirmed that our results were not explained by genetic confounding. Third, multiple sensitivity and complementary analyses yielded supporting evidence for our main conclusion. Fourth, the investigation of protein-cognition associations is relatively unexplored and has potentially wide-reaching implications for the general population. Finally, the genetic association of LRP8 with cognition demonstrates that this approach can recapitulate known biology.

There are also limitations to consider. First, our results may be biased by pleiotropic effects of the genetic variants on biological pathways that are independent of protein levels. The second key limitation is that the present analysis does not inform us on whether interventions on the levels of these proteins may influence cognitive performance. For instance, the protein levels may only be relevant during certain time windows of neurodevelopment, such as in the case of LRP8 which is known to influence neuronal development. In such a case, modifying protein levels in adulthood would not be expected to influence cognitive performance. Third, our findings warrant replication by leveraging gold-standard measures or different assays, since in this study a single SomaLogic aptamer assay was used to quantify relative protein abundance. In one possible source of bias in aptamer assays, missense variants that modify the amino acid sequence may consequently affect aptamer–protein binding without affecting the levels of the protein in the sample. Furthermore, aptamer-based protein quantification does not provide absolute protein quantities that are directly translatable to clinical settings. Our results should, therefore, be interpreted merely by their direction of the effect, rather than their effect size. Fourth, our analysis was restricted to individuals of European ancestry, so these findings may not accurately reflect the relationship between the CSF protein levels and cognitive function in diverse populations. Fifth, we did not identify effects of these proteins on the outcome of AD. This suggests that the direct effects of these proteins on AD are minimal, and any possible benefit on dementia risk would be mediated through mediated effects of improved cognitive reserve. Finally, the similarity of results across various tissue types precludes us from concluding that the CSF is the compartment in which these proteins influence cognition. In the setting of a similar genetic effect on protein abundance in different compartments, we are unable to resolve which of these biological compartments is more relevant to the mechanistic relationship between these proteins and cognition. Additional work is needed to identify the tissue type that is most relevant for these proteins to influence the respective phenotypes.

In conclusion, this MR analysis prioritizes the C1-esterase inhibitor and sTie-1 as proteins with potentially causal effects on cognitive performance. The biological mechanisms relating these proteins to cognition should be further investigated to determine which could be targeted to improve cognition.

## Data Availability

UK Biobank individual-level data used in this work can be accessed after applying for access at https://www.ukbiobank.ac.uk/enable-your-research/apply-for-access, accessed on 15 March 2020. All genetic data used in this work were obtained from publicly available sources. Link to datasets: Cerebrospinal fluid and plasma protein levels: https://www.niagads.org/datasets/ng00102, accessed on 10 February 2022; Cognitive performance and educational attainment: https://thessgac.com/papers/3, accessed on 10 February 2022; T1 MRI-derived phenotypes: https://open.win.ox.ac.uk/ukbiobank/big40/BIG40-IDPs_v4/IDPs.html, accessed on 10 February 2022; Reading and language-related skills phenotypes: https://www.genlang.org/downloads.html, accessed on 10 February 2022; Extremely high intelligence: http://ftp.ebi.ac.uk/pub/databases/gwas/summary_statistics/GCST005001-GCST006000/GCST005626/, accessed on 10 February 2022; General cognitive function phenotypes: https://www.ncbi.nlm.nih.gov/pmc/articles/PMC5974083/, accessed on 10 February 2022; Alzheimer’s disease: https://www.niagads.org/datasets/ng00075, accessed on 10 February 2022; Functional outcome after ischemic stroke: https://cd.hugeamp.org/downloads.html, accessed on 10 February 2022; Genotype–Tissue Expression data (GTEx): https://www.gtexportal.org/home/, accessed on 10 February 2022.

## Supplementary Materials

The following supporting information can be downloaded at: https://www.mdpi.com/article/10.3390/genes15010071/s1.
